# Differential Diagnosis of Retinal Vasculitis

**DOI:** 10.4103/0974-9233.58423

**Published:** 2009

**Authors:** Ahmed M. Abu El-Asrar, Carl P. Herbort, Khalid F. Tabbara

**Affiliations:** Department of Ophthalmology, College of Medicine, King Saud University, Riyadh, Saudi Arabia; 1 Inflammatory and Retinal Eye Diseases, Center for Ophthalmic Specialized Care and University of Lausanne, Lausanne, Switzerland; 2 The Eye Center and The Eye Foundation for Research in Ophthalmology, Riyadh, Saudi Arabia

**Keywords:** Fluorescein Angiography, Retina, Vasculitis

## Abstract

Retinal vaculitis is a sight-threatening inflammatory eye condition that involves the retinal vessels. Detection of retinal vasculitis is made clinically, and confirmed with the help of fundus fluorescein angiography. Active vascular disease is characterized by exudates around retinal vessels resulting in white sheathing or cuffing of the affected vessels. In this review, a practical approach to the diagnosis of retinal vasculitis is discussed based on ophthalmoscopic and fundus fluorescein angiographic findings.

## INTRODUCTION

Retinal vasculitis is a sight-threatening inflammatory eye condition that involves the retinal vessels. It may occur as an isolated idiopathic condition, as a complication of infective or neoplastic disorders, or in association with systemic inflammatory disease^1^ [Table 1].

**Table 1:** Disorders associated with retinal vasculitis^1^

Infectious disorders
Bacterial disorders
Tuberculosis, syphilis, lyme disease, Whipple's disease, brucellosis, cat scratch disease, endophthalmitis, post-streptococcal syndrome
Viral disorders
Human T cell lymphoma virus type 1, cytomegalovirus, herpes simplex virus, varicella zoster virus, Epstein-Barr virus, Rift Valley fever virus, hepatitis, acquired immunodeficiency syndrome, West Nile virus infection, Dengue fever virus
Parasitic disorders
Toxoplasmosis
Rickettsial disorders
Mediterranean spotted fever, Rocky Mountain spotted fever
Neurologic disorders
Multiple sclerosis
Microangiopathy of the brain, retina, and cochlea (Susac syndrome)
Malignancy
Paraneoplastic syndromes
Ocular lymphoma
Acute leukemia
Systemic inflammatory diseases
Behçet's disease
Sarcoidosis
Systemic lupus erythematosus
Wegener's granulomatosis
Polyarteritis nodosa
Churg-Strauss syndrome
Relapsing polychondritis
Slögren's A antigen
Rheumatoid arthritis
HLA-B27-associated uveitis
Crohn's disease
Postvaccination
Dermatomyositis
Takayasu's disease
Buerger's disease
Polymyositis
Ocular disorders
Frosted branch angiitis
Idiopathic retinal vasculitis, aneurysms, and neuroretinitis
Acute multifocal hemorrhagic retinal vasculitis
Idiopathic recurrent branch retinal arterial occlusion
Pars planitis
Birdshot chorioretinopathy

**Figure 1:**
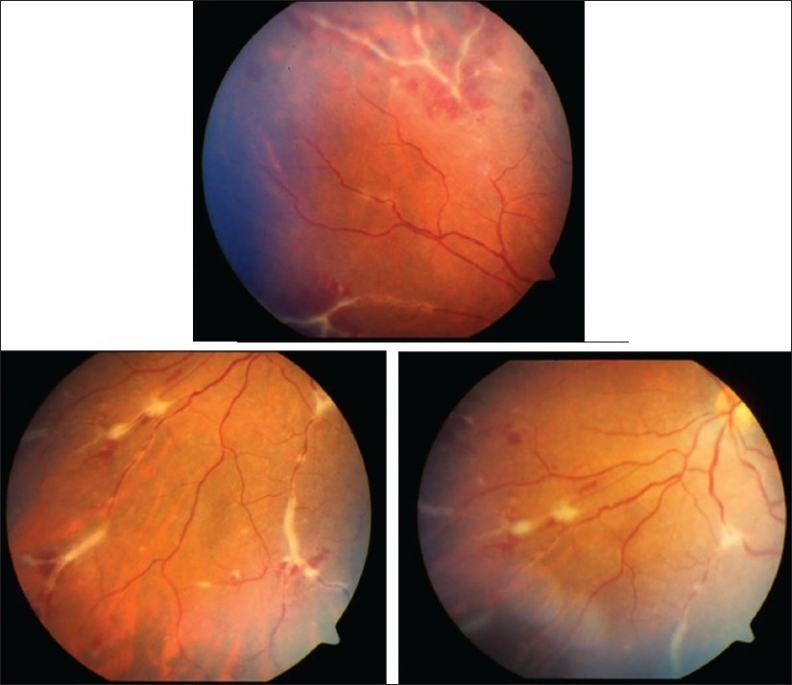
Fundus photographs of the left eye of a 27-year-old man with strongly positive tuberculin skin test demonstrating thick perivenous sheathing with intraretinal hemorrhages

Detection of retinal vasculitis is made clinically, and confirmed with the help of fundus fluorescein angiography. Active vascular disease is characterized by exudates around retinal vessels resulting in white sheathing or cuffing of the affected vessels, which may be segmental (skip lesions) or confluent [Figure 1].

Although retinal arterioles or branch retinal arteries may be involved in secondary systemic vasculitides such as systemic lupus erythematosus (SLE), as well as primary systemic vasculitides such as Wegener's granulomatosus, polyarteritis nodosa, Churg-Strauss syndrome or cryoglobulinemia,^2^ this usually leads to occlusion by microthrombosis, and intraocular inflammation is often not a feature of these diseases.^3^,^4^ Therefore, this type of occlusive vasculopathy should be recognized and distinguished from other conditions characterized by active vascular sheathing or cuffing with perivascular inflammatory infiltrate.

Retinal vasculitis results in leakage leading to retinal swelling, exudation, and macular edema. Cystoid macular edema is a significant contributing factor for poor vision in retinal vasculitis. Cystoid macular edema, when adequately treated with immunosuppressive therapy, is associated with a good prognosis.^5^ Occlusive retinal vasculitis affecting the retinal arterioles may cause cotton-wool spots representing microinfarcts of the retina. Central retinal artery and branch retinal artery occlusions are also reported in patients with retinal vasculitis.^6^–^12^ Occlusive periphlebitis can cause retinal edema, intraretinal hemorrhages, and hemorrhagic infarction of the retina. Poor visual outcome in some patients with retinal vasculitis, despite adequate therapy, may be explained by the presence of macular ischemia.^5^,^13^ Late changes secondary to vascular occlusion and remodeling include telangiectasis, microaneurysms, and ischemia-induced neovascularization, with sequelae such as recurrent vitreous hemorrhage, traction retinal detachment, rubeosis iridis, and neovascular glaucoma that can lead to functional loss of the eye.^6^,^14^–^20^ Inflammatory branch retinal vein occlusions are strongly associated with Behçet's disease and might contribute to visual loss.^21^–^23^

## FUNDUS FLUORESCEIN ANGIOGRAPHY

**Figure 2:**
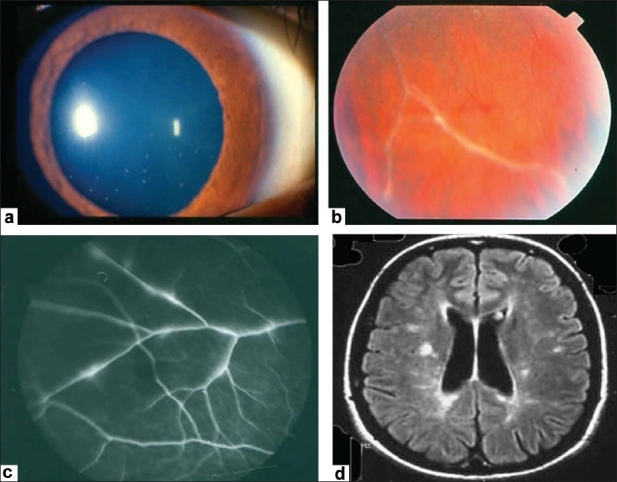
(a) Anterior segment of a 24-year-old woman with multiple sclerosis showing granulomatous keratic precipitates (b) Fundus photograph showing perivenous sheathing (c) Fluorescein angiogram showing focal venous leakage (d) Magnetic resonance imaging of the brain showing demyelinating lesions

**Figure 3:**
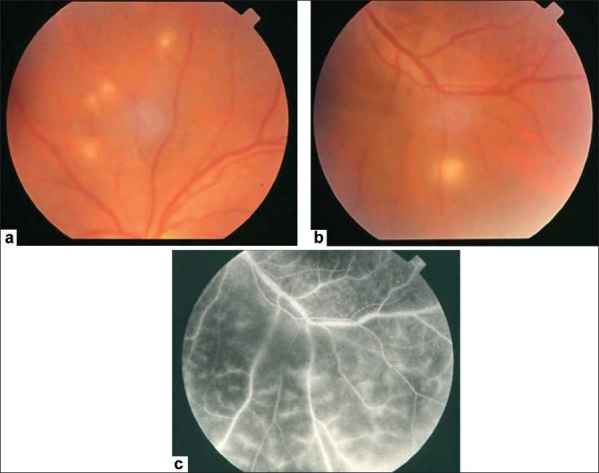
(a and b) Fundus photographs of a 22-year-old man with documented Behçet's disease showing characteristic multiple retinal infiltrates (c) Fluorescein angiogram showing characteristic extensive leakage from all retinal vessels

**Figure 4:**
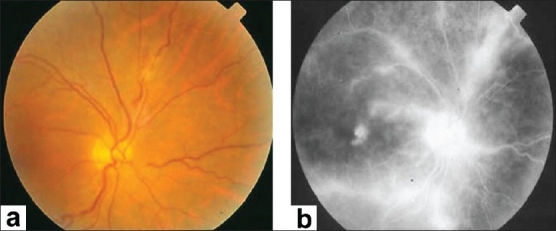
(a) Fundus photograph of the right eye of a 45-year-old woman with strongly positive tuberculin skin test demonstrating focal sheathing (b) Fluorescein angiogram showing extensive leakage from the retinal veins and optic nerve head

**Figure 5:**
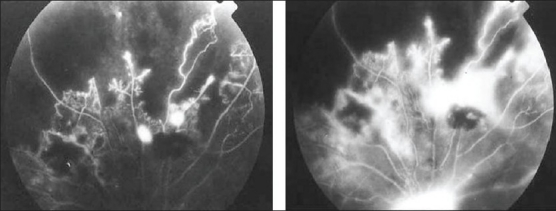
(a) Fluorescein angiogram of a 27-year-old man with strongly positive tuberculin skin test showing peripheral capillary nonperfusion, telangiectasia, abnormal vascular anastomosis, and neovascularization (b) The neovessels demonstrate extensive leakage

**Figure 6:**
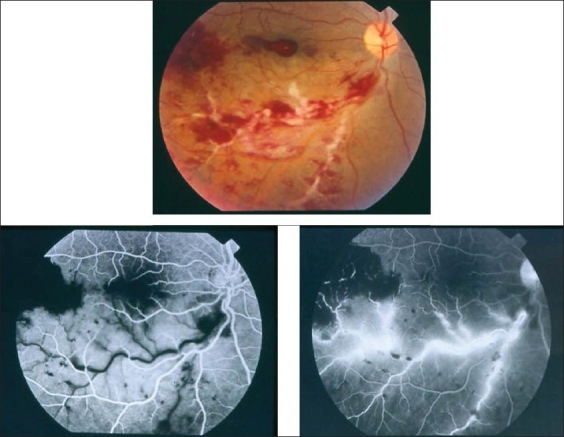
(a) Fundus photograph of the right eye of a 23-year-old man with documented Behçet's disease showing extensive perivenous sheathing involving inferotemporal vein with intraretinal hemorrhages and hemorrhagic infarction temporal to the macula (b) Fluorescein angiogram showing delayed filling of the involved vein (c) and late leakage and staining

Characteristic features seen with fluorescein angiography in active vasculitis include leakage of dye due to breakdown of the inner blood-retinal barrier, and staining of the blood vessel wall with fluorescein. Such leakage may be focal as seen in sarcoidosis or multiple sclerosis [Figure 2] or more diffuse, as seen in Behçet's disease and retinal vasculitis associated with tuberculoprotein hypersensitivity (Eales' disease) [Figures 3 and 4]. Diffuse capillary leakage is also a common finding in many conditions such as Behçet's disease and birdshot chorioretinopathy [Figure 3]. Fluorescein angiography is a more sensitive technique and will frequently show that the vasculitis is more extensive than the clinical examination suggests [Figure 4]. Fluorescein angiography is very useful to delineate areas of capillary nonperfusion, and neovascularization secondary to retinal ischemia [Figure 5]. It is also very valuable to diagnose the presence of inflammatory branch retinal vein occlusion [Figure 6].

**Figure 7:**
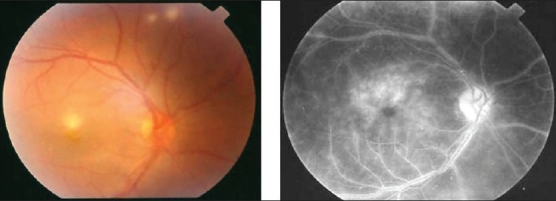
(a) Fundus photograph of the right eye of a 23-year-old man with documented Behçet's disease showing retinal infiltrates (b) Fluorescein angiogram showing leakage from retinal vessels and the multilobular pattern of cystoid macular edema

**Figure 8:**
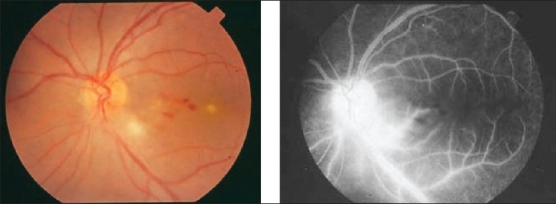
(a) Fundus photograph of the left eye of a 23-year-old man with documented Behçet's disease showing juxtapapillary retinal infiltrate and intraretinal hemorrhages (b) Fluorescein angiogram showing leakage from optic nerve head and retinal vessels

The ability to identify retinal vasculitis as ischemic by fluorescein angiography has important implications for management and is discussed later. Other angiographic findings include cystoid macular edema [Figure 7] and optic disc leakage [Figure 8]. Leakage of dye from the optic nerve head arises from dilated capillaries, which may be due to either to primary infiltration as in sarcoidosis^24^ or secondary vascular changes induced by intraocular inflammation.

In this review, we discuss a practical approach to the diagnosis of retinal vasculitis based on ophthalmoscopic and fluorescein angiographic findings [Table 2].

**Table 2:** Differential diagnosis of retinal vasculitis based on ophthalmoscopic findings

Ophthalmoscopic finding	Possible diagnoses
Phlebitis	Behçet's disease, tuberculosis, sarcoidosis, multiple sclerosis, pars planitis, Eales' disease, human immunodeficiency virus infection
Arteritis	Acute retinal necrosis, idiopathic retinal vasculitis, aneurysms and neuroretinitis (IRVAN), systemic vasculitides such as systemic lupus erythematosus (SLE), polyarteritis nodosa (PAN), Wegener's ganulomatosis, Churg-Strauss syndrome, and cryoglobulinemia
Cotton-wool spots	Systemic vasculitides such as SLE, PAN, Wegener's granulomatosis, Churg-Strauss syndrome, and cryoglobulinemia
Intraretinal infiltrates	Behçet’s disease, rickettsial infection, cat scratch disease
Necrotizing retinitis	Ocular toxoplasmosis, acute retinal necrosis, cytomegalovirus retinitis
Aneurysmal dilatations of the retinal and optic nerve head arterioles	IRVAN, sarcoidosis
Frosted branch angiitis	Idiopathic, infiltration with malignant cells (lymphoma or leukemia), SLE, Crohn's disease, toxoplasmic retinochoroiditis, human T cell lymphoma virus type 1 infection, acquired immunodeficiency syndrome, human immunodeficiency virus infection, herpes simplex virus infection, Epstein-Barr virus infection
Retinal ischemia	Tuberculosis, Eales' disease, Behçet’s disease, multiple sclerosis (rare), sarcoidosis (rare)
Inflammatory branch retinal vein occlusion	Behçet's disease, tuberculosis, sarcoidosis (rare)
Retinal arterial occlusions	SLE, PAN, Wegener's granulomatosis, Churg-Strauss syndrome, Crohn's disease, Susac syndrome, cat scratch disease, Mediterranean spotted fever, ocular toxoplasmosis

### Identification of retinal vessels involved

**Figure 9:**
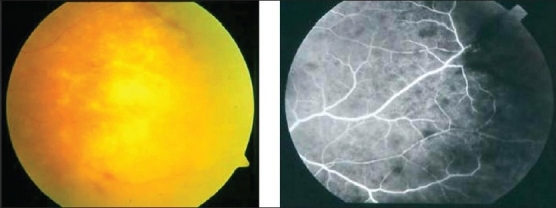
(a) Peripheral retina of a 30-year-old woman with acute retinal necrosis showing periarterial vascular sheathing and necrotizing retinitis (b) Fluorescein angiogram showing peripheral occlusive vasculopathy

Retinal vasculitis affecting predominantly the veins (phlebitis) has been described in association with Behçet's disease, tuberculosis, sarcoidosis, multiple sclerosis, pars planitis, retinal vasculitis associated with tuberculoprotein hypersensitivity (Eales' disease), human immunodeficiency virus infection (HIV) [Figures 1,2,4, 6]. Retinal arteritis is more commonly seen in acute retinal necrosis [Figure 9], idiopathic retinal vasculitis, aneurysms, and neuroretinitis (IRVAN) [Figure 10] and systemic vasculitides such as SLE, polyarteritis nodosa, and Wegener's granulomatosis, Churg-Strauss syndrome and cryoglobulinemia.^1^

**Figure 10:**
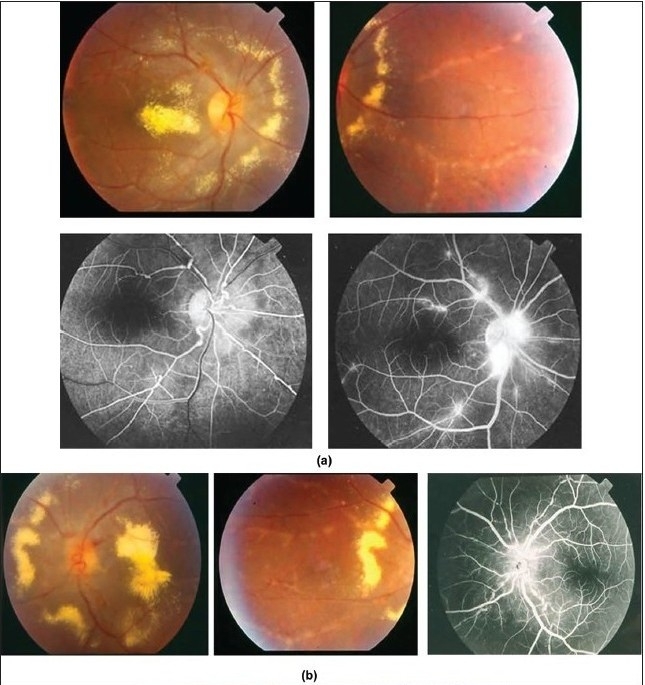
Fundus photographs of the right (a) and left; (b) eyes of a patient with idiopathic retinal vasculitis, aneurysms, and neuroretinitis showing extensive peripapillary and macular lipid exudates deposition, aneurysmal dilatations along the retinal arterioles, and perivascular sheathing affecting the retinal arterioles

**Figure 10c:**
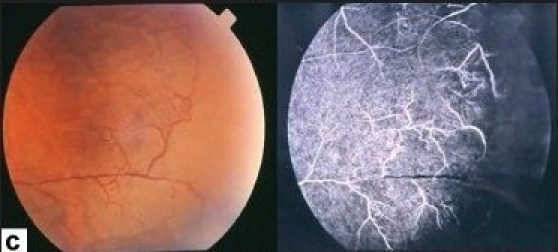
Fluorescein angiograms showing the aneurysmal changes along the retinal arterioles and peripheral nonperfusion

**Figure 10d:**
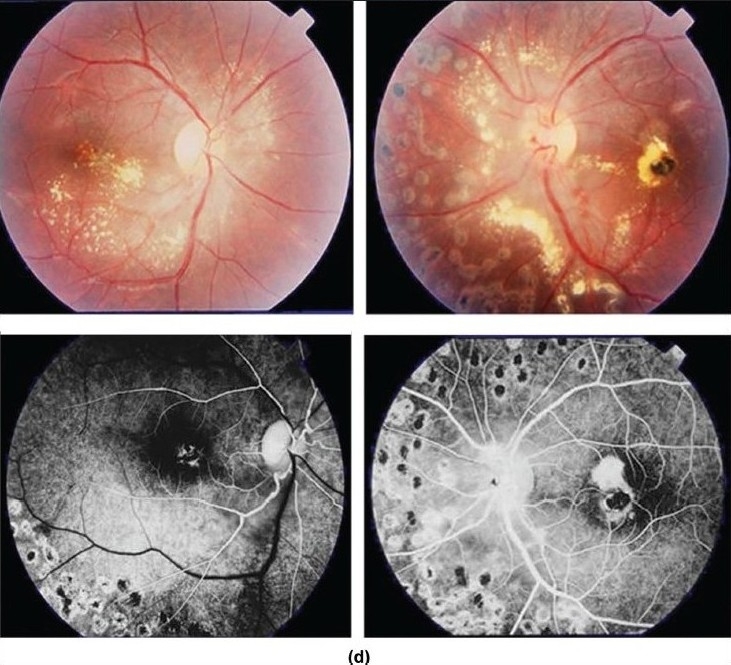
Fundus photographs 3 years after treatment with panretinal photocoagulation and systemic steroids showing regression of aneurismal dilatations and absorption of hard exudates

### Cotton-wool spots

**Figure 11:**
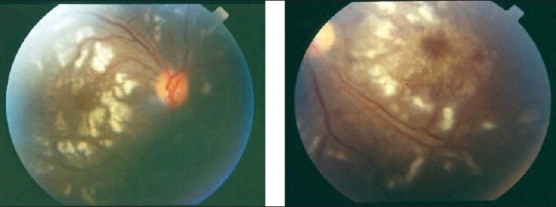
Fundus photographs of the (a) and (b) eyes of a 21-year-old woman with active systemic lupus erythematosus showing bilateral retinopathy consisting of multiple cotton-wool spots, and intraretinal hemorrhages

Cotton-wool spots representing microinfarcts of the retina due to precapillary retinal arteriolar occlusion are most often found in association with systemic vasculitides such as SLE, plyarteritis nodosa, Wegener's granulomatosis, Churg-Strauss syndrome, and cryoglobulinemia [Figure 11].

**Figure 12:**
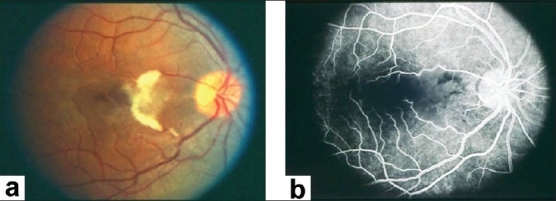
(a) Fundus photograph of the right eye of a 24-year-old woman with systemic lupus erythematosus and strongly positive lupus anticoagulant in whom increase in disease activity was manifested as a cilioretinal artery occlusion with ischemic retinal whitening in the area of the papillomacular bundle, indicating more extensive ischemia than that producing cotton-wool spots, as well as periarterial sheathing (b) Fluorescein angiogram showing occlusion of the cilioretinal artery; the area of retinal ischemia appears hypofluorescent and obscures the background choroidal flush

Retinal vascular lesions are the most common ophthalmic manifestations of SLE and are due to arterial occlusion. Retinopathy generally consists of cotton-wool spots with or without retinal hemorrhages and may occur in the absence of hypertension [Figure 11]. By contrast, a less common but more severe retinal vascular occlusive disease characterized by diffuse arteriolar occlusion with extensive capillary non-perfusion has been described.^18^ A more focal vascular disease, including retinal artery or vein occlusion may occur. Patients with SLE and raised antiphospholipid antibodies have a higher risk of developing occlusive retinal vascular disease.^25^ In addition, a patient with severe SLE-associated frosted branch periphlebitis and exudative maculopathy was reported.^26^ Exacerbations of disease activity might manifest only in the retina as a retinal vascular occlusion^7^ [Figure 12]. Retinal vascular involvement in polyarteritis nodosa is primarily arterial and gives rise to retinal vasculitis, cotton-wool spots, edema, hemorrhage, and central retinal artery occlusion. The disease may also involve choroidal vessels.^9^,^12^,^27^,^28^ Curi *et al*.^29^ reported aggressive retinal vasculitis involving both arteries and veins in a patient with polyarteritis nodosa.

Susac syndrome is a rare disease of unknown pathogenesis. It is also caused by microangiopathy affecting the arterioles of the brain, retina, and cochlea, giving the classic triad of encephalopathy, branch retinal arterial occlusions, and sensorineural hearing loss. The underlying process is believed to be a small vessel vasculitis causing microinfarcts of the retina, brain, and cochlea. Susac syndrome usually occurs in young women but can affect men. In those cases in which a brain biopsy was performed, histopathologic examination results showed microinfarcts, perivascular inflammatory infiltrates of small vessels consistent with an active small vessel angiitis. Magnetic resonance imaging of the brain often shows lesions suggestive of multiple sclerosis. Fluorescein angiography shows focal nonperfusion of retinal arterioles and arteriolar wall hyperfluorescence.^30^–^33^

### Intraretinal infiltrates

Intraretinal infiltrates are characteristic of infectious processes, but in the absence of these, they are pathognomonic of Behçet's disease [Figure 3,7 and 8]. These transient white patches of retinitis, often with small adjacent hemorrhages, are almost always seen in patients with active posterior Behçet's uveitis. Typically, they are silent on fundus fluorescein angiography.

### Necrotizing retinitis

**Figure 13:**
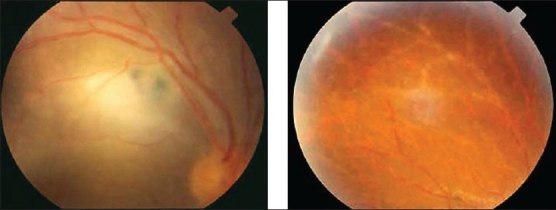
(a) Fundus photograph of the right eye showing toxoplasmic focal necrotizing retinitis adjacent to an old scar (b) Midperipheral retina showing extensive perivenous sheathing

Retinal vasculitis may be associated with necrotizing retinitis due to ocular toxoplasmosis, acute retinal necrosis, cytomegalovirus (CMV) retinitis, and rarely human T cell lymphoma virus type 1 (HTLV-1) associated uveitis. The hallmark of ocular toxoplasmosis is focal necrotizing retinitis, ultimately resulting in characteristic atrophic scars. Reactivation is frequently situated adjacent to an old atrophic scar with hyper pigmentation along the borders, indicating an old infection (satellite formation). Anterior uveitis, which may be granulomatous, and a secondary rise in intraocular pressure may also be noted. There may be an associated retinal vasculitis, which may be either near to or distant from the focus of active retinochoroiditis [Figure 13]. The arteries are often affected. Kyrieleis arterialitas refers to accumulation of periarterial exudates, which can occur either in the vicinity of the acute retinitis or elsewhere in the retina.^34^ In rare cases, the vasculitis may be occlusive, resulting in retinal infarction and consequent visual field defects. A case of frosted branch angiitis secondary to toxoplasmic retinochoroiditis was reported.^35^ In addition, Diaz-Valle *et al*.,^36^ reported a case of acute frosted branch angiitis without necrotizing chorioretinitis associated with acquired toxoplasmosis. The patient developed late peripheral retinochoroidal scar. Holland *et al*.^37^ reported the development of intraocular inflammatory reactions including vitritis, iridocyclitis, and retinal vasculitis without necrotizing retinal lesions in individuals with acquired systemic toxoplasmosis. Foci of retinitis or inactive retinochoroidal scars were seen in the same eyes during follow-up examinations suggesting that the initial inflammation may be caused by the presence of parasite in retinal tissue. These data strongly suggest that acquired systemic toxoplasmosis infection should be considered in the differential diagnosis of patients with retinal vasculitis, especially in the presence of constitutional symptoms suggesting systemic toxoplasmosis. More severe or atypical ocular presentations occur in immunocompromised patients.

Acute retinal necrosis is caused by viruses of the herpes group, mainly varicella zoster, herpes simplex types 1 and 2, and rarely, cytomegalovirus.^38^ The prominent features of acute retinal necrosis include peripheral necrotizing retinitis, retinal arteritis, and a prominent inflammatory reaction in the vitreous and anterior chamber [Figure 9]. Optic neuritis occurs in many affected eyes, and complicated rhegmatogenous retinal detachments are often encountered as a late sequela of the condition.^39^ The disease can progress rapidly with vision loss due to macular involvement, retinal detachment, or optic neuropathy. Vasculitis is predominantly affecting peripheral arteries with closure probably at the origin of the peripheral necrosis and is suspected to be due to antigen-antibody complexes [Figure 9].

In majority of the cases, cytomegalovirus (CMV) retinitis is a manifestation of acquired immunodeficiency syndrome (AIDS), with CMV retinitis being the most common ocular infection in these patients. The classic description of CMV retinitis is one of scattered yellow-white areas of necrotizing retinitis with variable degrees of hemorrhage and mild vitreous inflammation (“cottage cheese with catsup” or “pizza pie” retinopathy). The pathway of expanding lesions can be predicted by the appearance of venous sheathing or white dots distal to the leading edge. CMV retinitis is often accompanied by varying amounts of retinal vasculitis consisting primarily of perivenous sheathing.^40^,^41^ Frosted branch angiitis was described in patients with AIDS associated with small patches of CMV retinitis.^42^,^43^ Perivasculitis of the peripheral vessels involving veins more often than arteries was described in patients with AIDS. CMV retinitis was not seen in these patients. The vasculitis was thought to be a noninfectious retinopathy associated with AIDS.^44^,^45^ Fine *etal*.,^46^ reported a case of HIV-infected child with frosted branch angiitis without CMV retinitis that was refractory to specific anti-cytomegalovirus therapy. The angiitis only improved after subsequent treatment with systemic corticosteroids suggesting that the frosted branch angiitis in this patient was not attributed to CMV.

HTLV-1 infection is endemic in Japan, the Caribbean islands, and parts of Central Africa and South America. The major target cell of HTLV-1 is the CD4^+^*t*-cell. HTLV-1 infection is the established cause of adult*t*-cell leukemia/lymphoma (ATL), an aggressive malignancy of CD4^+^ lymphocytes; HTLV-1 associated myelopathy (HAM)/tropical spastic paraparesis (TSP), a demyelinating inflammatory disease of the spinal cord; and HTLV-1 uveitis (HU), defined as uveitis of undetermined etiology in an HTLV-1 carrier. Clinically, HU has been described as an acute granulomatous or nongranulomatous uveal reactions that were accompanied by vitritris and retinal vasculitis. The ocular disease was considered benign, resolving over weeks in response to corticosteroid treatment, with low incidence of complications and good visual prognosis. Gray-white, granular deposits scattered on the retinal vessels in the posterior pole were noted. Similar materials were also found to deposit on the vitreoretinal interface of the foveolar areas. In addition, retinal vasculitis with sheathing of retinal veins in the periphery was described in patients with HTLV-1-associated myelopathy.^47^–^49^ Nakao and Ohba^50^ reported three HTLV-1-positive Japanese teenagers presenting with extensive retinal periphlebitis resembling frosted branch angiitis. The retinal vascular disease responded poorly to systemic corticosteroids, had a smoldering course, and eventually resulted in diffuse chorioretinal degeneration. Levy-Clarke *et al*.,^51^ reported a patient with ATL presenting as a bilateral retinal vasculitis associated with necrotizing retinitis.

### Aneurysmal dilatations of the retinal and optic nerve head arterioles

IRVAN is a rare clinical entity characterized by bilateral retinal arteritis, numerous aneurysmal dilatations of the retinal and optic nerve head arterioles, peripheral retinal vascular occlusion, neuroretinitis, and uveitis [Figure 10]. This syndrome typically affects young healthy individuals, has a female predominance, and is not associated with any systemic abnormalities. Visual loss is due to exudative maculopathy, and neovascular sequelae of retinal ischemia.^52^–^54^ Recently, we reported a patient who presented with features typical of IRVAN in whom medical evaluation disclosed allergic fungal sinusitis.^55^ Several reports described resolution of aneurysmal dilatations of the retinal arterioles in patients with IRVAN treated with systemic steroids and peripheral retinal photocoagulation [Figure 10].^55^–^58^ Recently, Samuel *et al*.^54^ recommended early panretinal laser photocoagulation when angiographic evidence of widespread retinal nonperfusion is present, and before (or shortly after) the development of neovascularization. Arterial macroaneurysms, occurring in elderly female patients with sarcoidosis associated with peripheral multifocal chorioretinitis have been described. These patients had severe cardiovascular disease.^59^–^61^

### Frosted branch angiitis

**Figure 14:**
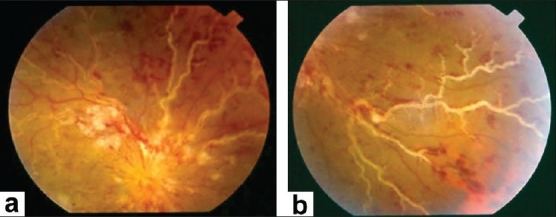
(a and b) Fundus photographs of a patient with clinical diagnosis of frosted branch angiitis showing prominent sheathing of the retinal veins, scattered intraretinal hemorrhages, cotton-wool spots, and optic disc swelling and hyperemia

**Figure 15:**
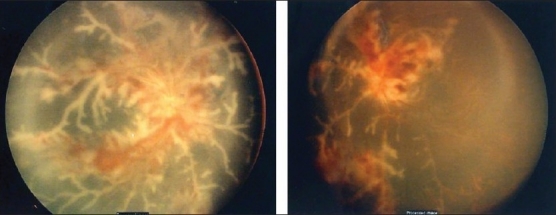
Fundus photograph of the right eye of a 19-year-old male with documented acute lymphoblastic leukaemia showing extensive sheathing of all retinal vessels producing frosted branch-like appearance (courtesy of E. Al-Kahtani, MD, Riyadh, Saudi Arabia)

Frosted branch angiitis (first described in 1976 by Ito *et al*.),^62^ occurs in young, healthy individuals who typically have acute bilateral (but sometimes unilateral) visual loss, associated with anterior and posterior segment inflammation. The retinal findings include swelling of the retina and severe sheathing of the retinal venules, creating the appearance of frosted tree branches. Additional findings include intraretinal hemorrhages, hard exudates, and serous exudative detachments of the macula and periphery [Figure 14]. Fluorescein angiography demonstrates leakage of dye from the vessels, but no evidence of decreased blood flow or occlusion. The disease usually responds rapidly to systemic corticosteroids with rapid resolution of the vascular sheathing. The visual prognosis is usually good and there is no recurrence in most patients. The term "acute frosted retinal periphlebitis" was suggested to describe the condition by Kleiner *et al*.^63^ Kleiner^64^ classified the patients with the appearance of frosted branch angiitis into three subgroups-First are patients with lymphoma or leukemia^64^ whose disease is due to infiltration with malignant cells (frosted branch-like appearance) [Figure 15] Second is the group of patients who have associated viral infections or autoimmune disease. Frosted branch angiitis was reported in patients with systemic lupus erythematosus,^26^ Crohn's disease,^65^ toxoplasmic retinochoroiditis,^35^,^36^ human T cell lymphoma virus type 1 infection,^50^ AIDS associated with small patches of retinitis,^42^,^43^ HIV without CMV retinitis,^46^ herpes simplex virus infection,^66^ and Epstein-Barr virus infection.^67^ In these patients, frosted branch angiitis is a clinical sign, possibly of immune complex deposition (secondary frosted branch angiitis). Finally, there is the group of otherwise healthy young patients described initially (acute idiopathic angiitis). It is likely that the frosted branch angiitis that developed in these patients represents an immune reaction to a number of different stimuli.^68^ A case of frosted branch angiitis complicated by bilateral retinal and optic nerve head neovascularization secondary to severe peripheral retinal ischemia was reported. 69 In addition, we reported the unusual association between severe retinal periphlebitis resembling frosted branch angiitis and nonperfused central retinal vein occlusion.^20^

### Retinal ischemia

Patients with ischemic retinal vasculitis represent a major management problem. It is important to identify the presence of retinal ischemia in patients with retinal vasculitis because panretinal laser photocoagulation should be considered when angiographic evidence of widespread retinal nonperfusion is present, and before (or shortly after) the development of neovascularization.

Ischemic retinal vasculitis is frequently seen secondary to tuberculosis and retinal vasculitis associated with tuberculoprotein hypersensitivity (Eales' disease) which is typically an obliterative periphlebitis affecting the retina in multiple quadrants, starting at or anterior to the equator and progressing posteriorly. Occasionally, it can begin close to the optic nerve head, mimicking a vein occlusion. Ophthalmoscopic findings vary and depend on the stage of the disease. Initially, it presents as active retinal periphlebitis with thick exudates around the retinal veins associated with retinal hemorrhages, and hemorrhagic infarctions of the retina [Figure 1,4,16–18]. Healed periphlebitis results in sclerosed white venules, and abnormal vascular anastomosis [Figure 19]. The periphlebitis may cause nonperfusion of a substantial portion of the retina that may lead to proliferative vascular retinopathy with sequelae such as recurrent vitreous hemorrhage, traction retinal detachment, rubeosis iridis, and neovascular glaucoma^1^,^49^,^70^–^73^ [Figures 5, 17–22]. The management of tuberculous retinal vasculitis or retinal vasculitis associated with tuberculoprotein hypersensitivity (Eales' disease) requires the use of systemic steroids and appropriate antituberculous therapy. New vessel formation associated with retinal vasculitis and capillary closure responds to panretinal photocoagulation [Figure 21]. Early vitrectomy and adequate endolaser photocoagulation should be considered in eyes with non-resolving vitreous hemorrhage associated with active fibrovascular proliferation^14^ [Figure 22].

**Figure 16:**
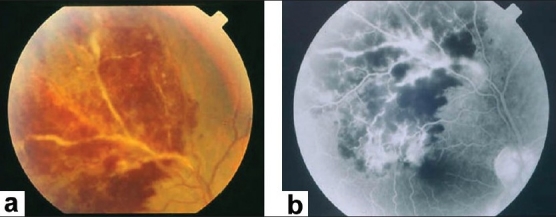
(a) Fundus photograph of the right eye of a 25-year-old man with strongly positive tuberculin skin test demonstrating thick perivenous sheathing with intraretinal hemorrhages and hemorrhagic infarction (b) Fluorescein angiogram showing leakage and staining of the involved veins

**Figure 17:**
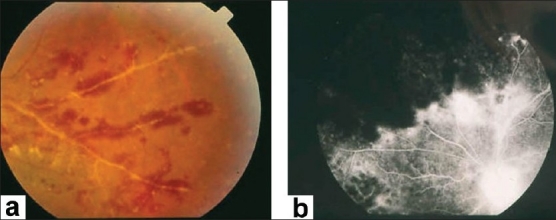
(a) Fundus photograph of the right eye of a 24-year-old man with strongly positive tuberculin skin test demonstrating thick perivenous sheathing and intraretinal hemorrhages (b) Fluorescein angiogram showing retinal nonperfusion

**Figure 18:**
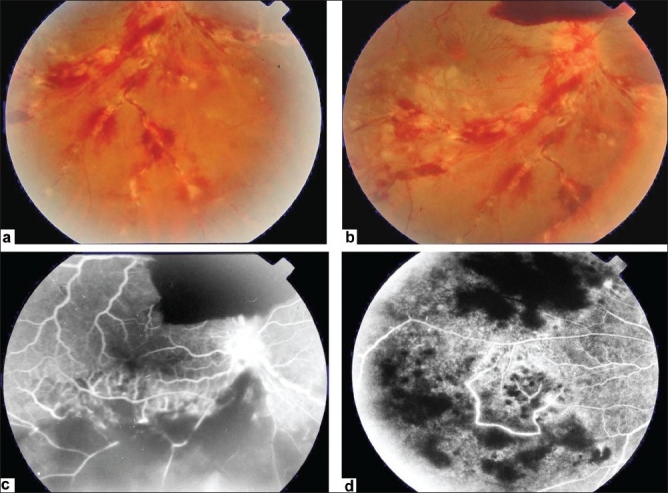
(a and b) Fundus photograph of the right eye of a 28-year-old man with strongly positive tuberculin skin test demonstrating thick perivenous sheathing; with intraretinal hemorrhages and preretinal hemorrhage above optic nerve head (c and d) Fluorescein angiogram showing leakage from the retinal veins, and neovessels on optic nerve head and retinal nonperfusion

**Figure 19:**
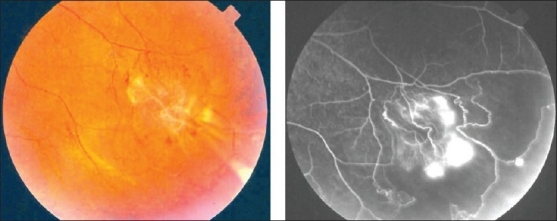
Fundus photograph of a 27-year-old man with strongly positive tuberculin skin test demonstrating sclerosed white retinal vessels in the periphery and neovessels Fundus fluorescein angiogram showing peripheral capillary nonperfusion, and leakage from neovessels

**Figure 20:**
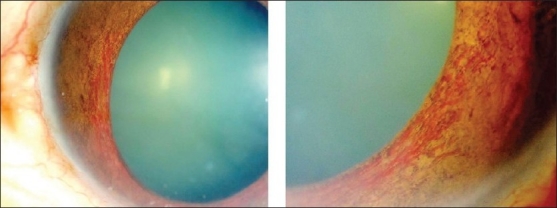
Anterior segment of a 30-year-old man with strongly positive tuberculin skin test showing extensive rubeosis iridis. This patient had, in addition, vitreous hemorrhage, retinal ischemia and retinal neovessels

**Figure 21:**
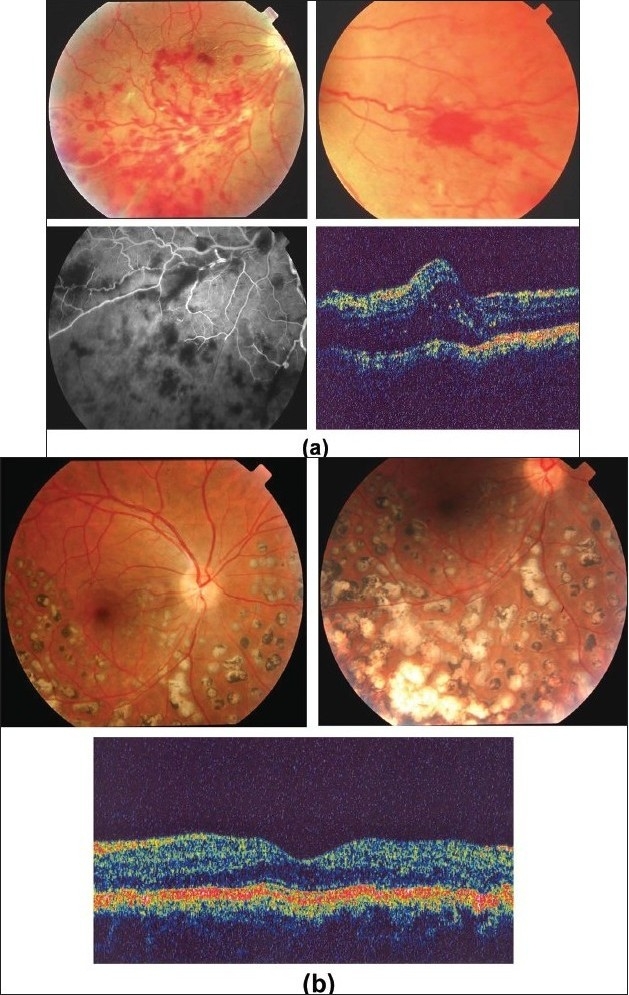
(a) Fundus photographs of the right eye of a 25-year-old man with strongly positive tuberculin skin test demonstrating perivenous sheathing with intraretinal hemorrhages (upper left) and neovessels nasal to optic nerve head (upper right) Fluorescein angiography showing retinal nonperfusion (bottom left) Optical coherence tomography showing macular edema (bottom right) (b) Fundus photographs after treatment with systemic steroids, appropriate antituberculous therapy, and scatter laser photocoagulation (upper left and right) showing resolution of perivenous sheathing and intraretinal hemorrhages and involution of neovessels Optical coherence tomography displays normal anatomy of the macula (bottom)

**Figure 22:**
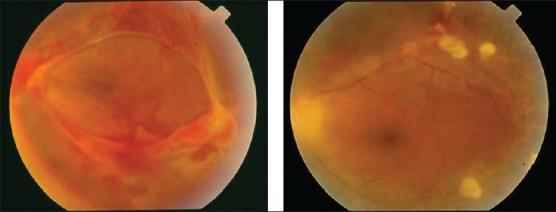
Fundus photograph of the left eye of a 32-year-old man with strongly positive tuberculin skin test showing vitreous hemorrhage and active fibrovascular tissue on the optic nerve head and along the vascular arcades (left) Fundus photograph after pars plana vitrectomy and endolaser photocoagulation (right) showing clear vitreous cavity and involution of neovessels

**Figure 23:**
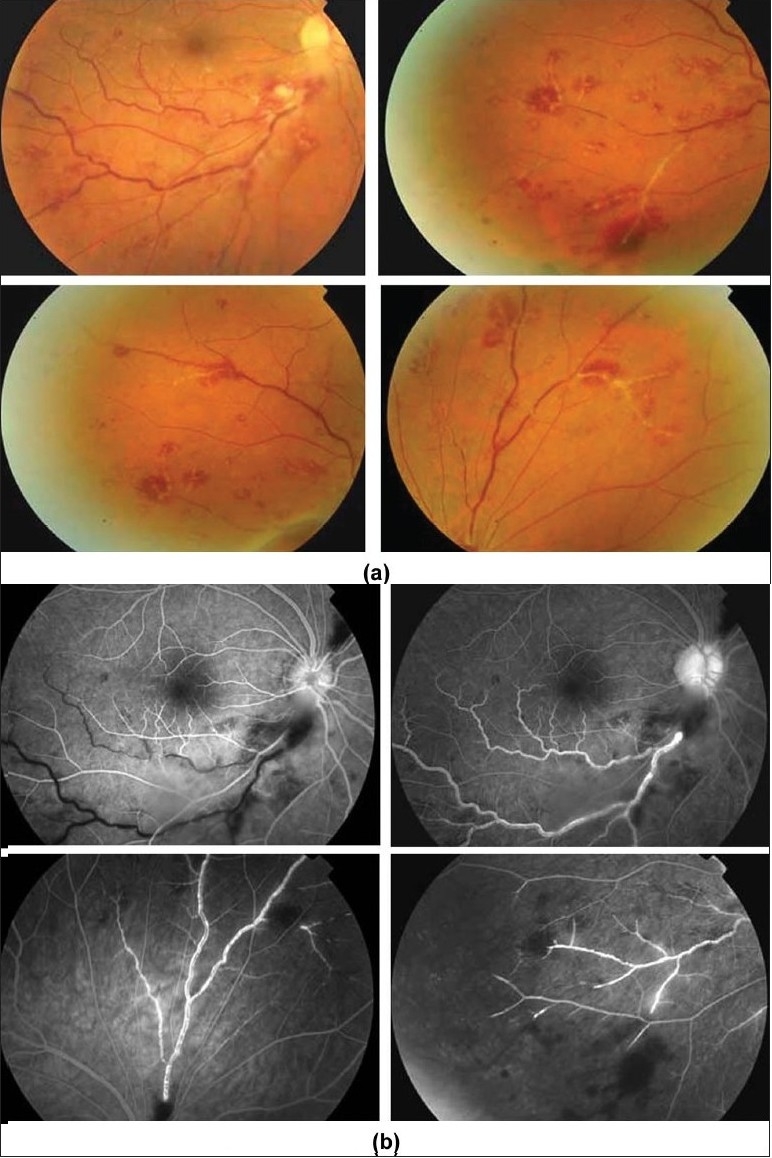
(a) Fundus photographs of the right eye of a 27-year-old man with documented Behçet’s disease showing perivenous sheathing, intraretinal hemorrhages, and retinal infiltrates (b) Fluorescein angiogram showing delayed filling of the inferotemporal vein (upper left), late leakage and staining of the involved veins, and retinal nonperfusion (bottom left)

Ischemic retinal vasculitis may also be secondary to Behçet's disease and multiple sclerosis. The diagnosis is made using criteria proposed by the International Study Group for Behçet's disease in 1990.^74^ The criteria require recurrent oral ulceration as an essential symptom plus any two or more symptoms of genital ulceration, eye lesions, skin lesions and a positive pathergy test to make a diagnosis of Behçet's disease. Inflammatory eye disease generally appears later than the oral ulceration and develops in about 70% of patients in Japan. The ocular manifestations of Behçet's disease typically include recurrent attacks of anterior uveitis, with or without hypopyon, cellular infiltration and opacification of the vitreous, retinal vasculitis [Figures 6 and 23], retinal infiltrates [Figures  3, 7 and 8] and hemorrhages, cystoid macular edema [Figure 8] and disc hyperemia. Retinal vasculitis and recurrent vasoocclusive episodes are the major causes of visual morbidity. In addition, inflammatory retinal vein occlusions are strongly associated with Behçet's disease^21^–^23^,^75^] [Figures 6 and 23]. Fluorescein angiography may show diffuse retinal vascular leakage, late staining of the vasculature, leakage from the disc, macular edema, areas of capillary dropout, and neovascularization [Figures 3, 6–8 and 23].

Various ocular inflammatory changes have been described in patients with multiple sclerosis and may be the presenting sign of the disease. They include nongranulomatous and granulomatous iridocyclitis, intermediate uveitis, retinitis, periphlebitis [Figure 2] and optic neuritis.^15^–^17^,^76^–^83^ Retinal periphlebitis has been described as a common manifestation of multiple sclerosis. It has been observed with an average frequency of 11.5% in more than 3000 published cases of MS examined for sheathing.^79^ In an autopsy series of 93 eyes from patients with definite multiple sclerosis, segmental lympho-plasmacytic perivenous infiltrates were found in seven eyes and focal lymphocytic or granulomatous retinitis was present in five eyes.^76^ The foci of granulomatous retinal inflammatory cells were noted in the inner retina and overlying vitreous and corresponded to white plaques visible on the inner retinal surface on gross examination.^76^ Round dot-like opacities, the diameter of a medium-sized vein, visible in the vitreous immediately overlying the retina were originally described by Rucker.^81^ These have come to be known as “Rucker bodies.” It has been demonstrated that demyelinative plaques in the brain typically encircle a venule and that in an active lesion perivenular infiltrates are present.^82^ These changes appear similar to the periphlebitis occurring in the retina. The periphlebitis can progress to occlusive peripheral vasculitis, which results in peripheral retinal neovascularization and tractional or rhegmatogenous retinal detachments or both. Peripheral scatter photocoagulation and vitrectomy may be required to stabilize the proliferative retinopathy.^15^–^17^,^83^

**Figure 24:**
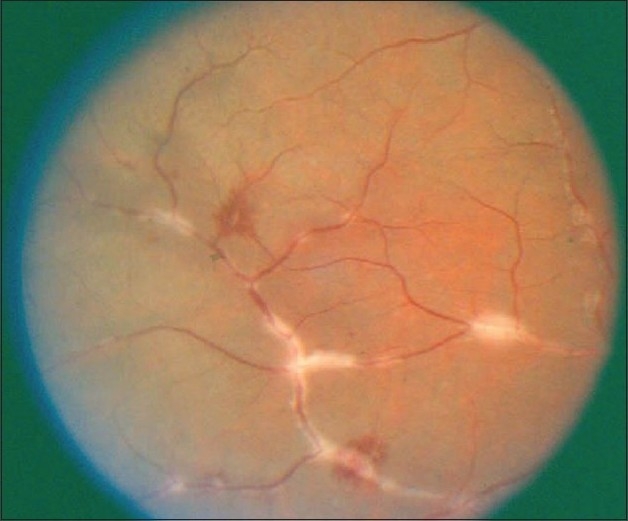
Fundus photograph of a patient with documented sarcoidosis demonstrating segmental perivenous sheathing

Retinal periphlebitis associated with sarcoidosis is usually nonocclusive, sometimes subclinical and only visible on fluorescein angiography, associated with typical segmental cuffing or more extensive sheathing and perivenous exudates, which are usually indicated as “candle wax drippings” [Figure 24]. Multiple small round chorioretinal lesions are frequently seen in peripheral fundus. Peripheral multifocal chorioretinitis and choroidal granuloma were described. Optic disc swelling may be caused by uveitis, raised intracranial pressure, or optic nerve infiltration. Arterial macroaneurysms, occurring in elderly female patients with peripheral multifocal chorioretinitis have been described, and were associated with severe cardiovascular disease.^84^,^85^ Development of capillary nonperfusion, and subsequent neovascularization as well as branch and central retinal vein occlusions have been described.^86^–^89^

**Figure 25:**
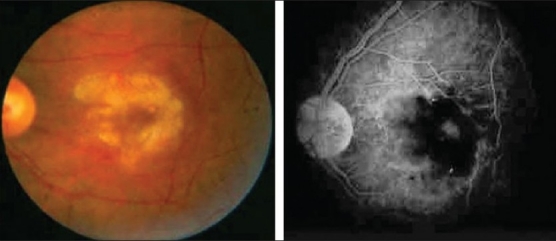
Fundus photograph of a patient with rift valley fever virus infection showing retinitis in the macular area (left); Fluorescein angiogram showing occlusive retinal vasculitis (right) (Courtesy of E. Abboud, MD, Riyadh, Saudi Arabia)

## OTHER CAUSES OF OCCLUSIVE RETINAL VASCULITIS

Idiopathic recurrent branch retinal arterial occlusion is a clinical syndrome characterized by recurrent multiple branch retinal arterial occlusions of unknown cause in one or both eyes of healthy middle-aged patients. Ophthalmoscopic and fluorescein angiographic findings suggested focal arteritis and arteriolitis as the cause of the obstructions. Eyes that have large areas of retinal ischemia may subsequently develop preretinal neovascularization. The prognosis for maintaining good visual acuity is good. Detailed investigation of these patients has failed to reveal a systemic etiology. Vestibuloauditory and/or transient sensorimotor symptoms were detected in 50% of patients. It is postulated that many of the patients have mild or partial manifestations of the microangiopathic syndrome of encephalopathy, hearing loss, and retinal arteriolar occlusions.^11^ Occlusive retinal vasculitis, and subsequent retinal neovascularization^6^ and neovascular glaucoma^19^ were also reported in patients with Crohn's disease. Occlusive retinal vasculitis has also been reported in patients infected with Rift Valley fever virus^90^,^91^ [Figure 25] and West Nile virus.^92^–^94^

## DIAGNOSTIC EVALUATION

The search for a cause in patients with retinal vasculitis often involves a multidisciplinary approach and laboratory investigation^2^,^95^ [Table 3]. Discrimination between infectious or noninfectious etiology of retinal vasculitis is important because treatment is different. Immunosuppressive therapy may be essential in certain disorders but it might be deleterious in infectious entities. Once an infectious cause is believed to be unlikely, an associated systemic disease should be considered and an appropriate investigation instituted. In cases of diagnostic doubt, malignancy must be ruled out and should certainly be considered if, after an initial improvement with therapy, the patient's disease rapidly becomes refractory to treatment. The ophthalmologist, therefore, has a major role in clarifying the nosologic and diagnostic debate in patients with retinal vasculitis.

**Table 3:** Diagnostic studies performed on patients with retinal vasculitis*

Laboratory tests
Complete blood count with differential
Erythrocyte sedimentation rate
C-reactive protein
Serum chemistry panel with tests for renal and liver functions
Blood sugar
Urinalysis
Venereal Disease Research Laboratory (VDRL) test, Fluorescent treponemal antibody absorption (FTA-ABS) test
Tuberculin skin testing
Gamma interferon release assays for tuberculosis
Toxoplasmosis serology
Lyme disease serology
Dengue virus serology
Cat scratch disease serology
Rickettsial serology
Human immunodeficiency virus, human T cell lymphoma virus type1, cytomegalovirus, herpes simplex virus, varicella zoster virus, hepatitis virus, and West Nile virus serology
Polymerase chain reaction to identify pathogens in ocular specimens
Serum angiotensin-converting enzyme
Rheumatoid factor
Antinuclear antibody
Anti-dsDNA
Antineutrophil cytoplasmic antibody
Antiphospholipid antibodies (lupus anticoagulants and anticardiolipin antibodies)
Serum complement, CH50, AH50
Extractable nuclear antigen
Serum protein electrophoresis
Serum cryoglobulins
Human leukocyte antigen testing
Vitreous biopsy
Cerebrospinal fluid cytology and cell count
Imaging
Fluorescein angiography
Optical coherence tomography
Ultrasonography
Chest X-ray
CT scanning
Magnetic resonance imaging
Gallium scan
Sacroiliac X-ray

The diagnostic work-up should be tailored according to the patient’s medical history, review of systems, and physical examination.^1^

The laboratory work-up of a patient with retinal vasculitis should be based on a differential diagnosis derived from a detailed history, review of systems, and physical examination. If the patient's medical history, review of systems, or ocular examination suggests an underlying systemic disease, then the diagnostic work-up should be tailored for that disease. The absence of any diagnostic clues from history makes idiopathic retinal vasculitis most likely. If, however, the patient has no signs or symptoms suggestive of an associated disease then the work-up of the patient is limited to a fluorescein angiogram, complete blood count, erythrocyte sedimentation rate, VDRL, FTA-ABS, blood chemistry, urinalysis, tuberculin skin testing, HIV serology, and chest radiograph. Numerous studies have shown that little additional information is gained by “blind” investigation of the patient and that pursuing this is neither time nor cost effective.

## References

[1] Abu El-Asrar AM, Herbort CP, Tabbara KF (2005). Retinal vasculitis. Ocul Immunol Inflamm.

[2] Perez VL, Chavala SH, Ahmed M, Chu D, Zafirakis P, Baltatzis S (2004). Ocular manifestations and concepts of systemic vasculitides. Surv Ophthalmol.

[3] Graham EM, Spalton DJ, Barnard RO, Garner A, Russell RW (1985). Cerebral and retinal vascular changes in systemic lupus erythematosus. Ophthalmology.

[4] Au A, O'Day J (2004). Review of severe vaso-occlusive retinopathy in systemic lupus erythematosus and the antiphosopholipid syndrome: Associations, visual outcomes, complications and treatment. Clin Exp Ophthalmol.

[5] Palmer HE, Stanford MR, Sanders MD, Graham EM (1996). Visual outcome of patiets with idiopathic ischaemic and non-ischaemic retinal vasculitis. Eye.

[6] Saatci OA, Koçak N, Durak I, Ergin MH (2001). Unilateral retinal vasculitis, branch retinal artery occlusion and subsequent retinal neovascularization in Crohn's disease. Int Ophthalmol.

[7] el-Asrar AM, Naddaf HO, Al-Momen A, Al-Balla SR (1995). Systemic lupus erythematosus flare-up manifesting as a cilioretinal artery occlusion. Lupus.

[8] Iida T, Spaide RF, Kantor J (2002). Retinal and choroidal arterial occlusion in Wegener's granulomatosis. Am J Ophthalmol.

[9] Hsu CT, Kerrison JB, Miller NR, Goldberg MF (2001). Choroidal infarction, ischemic optic neuropathy, and central retinal artery occlusion from polyarteritis nodosa. Retina.

[10] Skrapari I, Kagkelari E, Charitatos E, Pantelidaki C, Gounaris T, Sioula E (2008). Acute painless monocular visual loss due to central retinal artery occlusion in a patient with Churg-Strauss vasculitis. Clin Rheumatol.

[11] Johnson MW, Thomley ML, Huang SS, Gass JD (1994). Idiopathic recurrent branch retinal arterial occlusion: Natural history and laboratory evaluation. Ophthalmology.

[12] Emad Y, Basaffar S, Ragab Y, Zeinhom F, Gheita T (2007). A case of polyarteritis nodosa complicated by left central retinal artery occlusion, ischemic optic neuropathy, and retinal vasculitis. Clin Rheumatol.

[13] Bentley CR, Stanford MR, Shilling JS, Sanders MD, Graham EM (1993). Macular ischaemia in posterior uveitis. Eye.

[14] el-Asrar AM, AL-Kharashi SA (2002). Full panretinal photocoagulation and early vitrectomy improve prognosis of retinal vasculitis associated with tuberculoprotein hypersensitivity (Eales' disease). Br J Ophthalmol.

[15] Vine AK (1992). Severe periphlebitis, peripheral retinal ischemia, and preretinal neovascularization in patients with multiple sclerosis. Am J Ophthalmol.

[16] Morse PH (1975). Retinal venous sheathing and neovascularization in disseminated sclerosis. Ann Ophthalmol.

[17] Valentincic NV, Kraut A, Rothova A (2007). Vitreous hemorrhage in multiple sclerosis-associated uveitis. Ocul Immuno Inflamm.

[18] Jabs DA, Fine SL, Hochberg MC, Newman SA, Heiner GG, Stevens MB (1986). Severe retinal vaso-occlusive disease in systemic lupus erythematosus. Arch Ophthalmol.

[19] Salmon JF, Ursell PG, Frith P (2000). Neovascular glaucoma as a complication of retinal vasculitis in Crohn's disease. Am J Ophthalmol.

[20] Abu El-Asrar AM, Al-Obeidan SA, Abdel Gader AGM (2003). Retinal periphlebitis resembling frosted branch angiitis with nonperfused central retinal vein occlusion. Eur J Ophthalmol.

[21] Sakane T, Takeno M (2000). Novel approaches to Behçet's disease. Exp Opin Invest Drugs.

[22] Okada AA (2000). Drug therapy in Behçet's disease. Ocul Immunol Inflamm.

[23] Graham EM, Stanford MR, Sander MD, Kasp E, Dumonde DC (1989). A point prevalence study of 150 patients with idiopathic retinal vasculitis: I: Diagnostic value of ophthalmological features. Br J Ophthalmol.

[24] Gass JD, Olson CL (1976). Sarcoidosis with optic nerve and retinal involvement. Arch Ophthalmol.

[25] Asherson RA, Merry P, Acheson JF, Harris EN, Hughes GR (1989). Antiphospholipid antibodies: A risk factor for occlusive ocular vascular disease in systemic lupus erythematosus and the primary antiphospholipid syndrome. Ann Rheum Dis.

[26] Quillen DA, Stathopoulos NA, Blankenship GW, Ferriss JA (1997). Lupus associated frosted branch periphlebitis and exudative maculopathy. Retina.

[27] Morgan CM, Foster CS, D'Amico DJ, Gragoudas ES (1986). Retinal vasculitis in polyarteritis nodosa. Retina.

[28] Akova YA, Jabbur NS, Foster CS (1993). Ocular presentation of polyarteritis nodosa: Clinical course and management with steroid and cytotoxic therapy. Ophthalmology.

[29] Curi AL, Freeman G, Pavesio C (2001). Aggressive retinal vasculitis in polyarteritis nodosa. Eye.

[30] O'Halloran HS, Pearson PA, Lee WB, Susac JO, Berger JR (1998). Microangiopathy of the brain, retina, and cochlea (Susac syndrome): A report of five cases and a review of the literature. Ophthalmology.

[31] Do TH, Fisch C, Evoy F (2004). Susac syndrome: Report of four cases and review of the literature. AJNR Am J Neuroradiol.

[32] Susac JO, Egan RA, Rennebohm RM, Lubow M (2007). Susac's syndrome: 1975-2005 microangiopathy/autoimmune endotheliopathy. J Neurol Sci.

[33] Martinet N, Fardeau C, Adam R (2007). Fluorescein and indocyanine green angiographies in susac syndrome. Retina.

[34] Theodossiodis P, Kokolakis S, Ladas I, Kollia AC, Chatzoulis D, Theodossiadis G (1995). Retinal vascular involvement in acute toxoplasmic retinochoroiditis. Int Ophthalmol.

[35] Ysasaga JE, Davis J (1999). Frosted branch angiitis with ocular toxoplasmosis. Arch Ophthalmol.

[36] Diaz-Valle D, Diaz-Rodriguez E, Diaz-Valle T, Benítez del Castillo JM, Toledano N, Fernández Aceñero MJ (2003). Frosted branch angiitis and late peripheral retinochoroidal scar in a patient with acquired toxoplasmosis. Eur J Ophthalmol.

[37] Holland GN, Muccioli C, Silveira C, Weisz JM, Belfort R, O'Connor GR (1999). Intraocular inflammatory reactions without focal necrotizing retinochoroiditis in patients with acquired systemic toxoplasmosis. Am J Ophthalmol.

[38] Ganatra JB, Chandler D, Santos C, Kuppermann B, Margolis TP (2000). Viral causes of the acute retinal necrosis syndrome. Am J Ophthalmol.

[39] Holland GN; Executive Committee of the American Uveitis Society (1994). Standard diagnostic criteria for the acute retinal necrosis syndrome. Am J Ophthalmol.

[40] Yoser SL, Forster DJ, Rao NA (1993). Systemic viral infections and their retinal and choroidal manifestations. Surv Ophthalmol.

[41] Culbertson WW (1989). Infections of the retina in AIDS. Int Ophthalmol Clin.

[42] Spaide RF, Vitale AT, Toth IR, Oliver JM (1992). Frosted branch angiitis associated with cytomegalovirus retinitis. Am J Ophthalmol.

[43] Geier SA, Nasemann J, Klauss V, Kronawitter U, Goebel FD (1992). Frosted branch angiitis in a patient withthe acquired immunodeficiency syndrome. Am J Ophthalmol.

[44] Kestelyn P, Lepage P, Van de Perre P (1985). Perivasculitis of the retinal vessels as an important sign in children with AIDS-related complex. Am J Ophthalmol.

[45] Kestelyn P, Van de Perre P, Rouvroy D, Lepage P, Bogaerts J, Nzaramba D (1985). A prospective study of the ophthalmologic findings in the acquired immune deficiency syndrome in Africa. Am J Ophthalmol.

[46] Fine HF, Smith JA, Murante BL, Nussenblatt RB, Robinson MR (2001). Frosted branch angiitis in a child with HIV infection. Am J Ophthalmol.

[47] Sasaki K, Morooka I, Inomata H, Kashio N, Akamine T, Osame M (1989). Retinal vasculitis in human t-lymphotropic virus type 1 associated myelopathy. Br J Ophthalmol.

[48] Nakao K, Ohba N (1993). Clinical features of HTLV-1 associated uveitis. Br J Ophthalmol.

[49] Nakao K, Ohba N (1996). HTLV-1 associated uveitis revisited:Characteristic grey-white, granular deposits on retinal vessels. Br J Ophthalmol.

[50] Nakao K, Ohba N (2003). Human t-cell lymphotropic virus type 1-associated retinal vasculitis in children. Retina.

[51] Levy-Clarke GA, Buggage RR, Shen D, Vaughen LO, Chan CC, Davis JL (2002). Human *t*-cell lymphotropic virus-type 1 associated t-cell leukemia/lymphoma masquerading as necrotizing retinal vasculitis. Ophthalmology.

[52] Kincaid J, Schatz H (1983). Bilateral retinal arteritis with multiple aneurysmal dilatations. Retina.

[53] Chang TS, Aylward W, Davis JL, Mieler WF, Oliver GL, Maberley AL (1995). Idiopathic retinal vasculitis, aneurysms, and neuro-retinitis. Ophthalmology.

[54] Samuel MA, Equi RA, Chang TS, Mieler W, Jampol LM, Hay D (2007). Idiopathic retinitis, vasculitis, aneurysms, and neuroretinitis (IRVAN): New observations and a proposed staging system. Ophthalmology.

[55] Abu El-Asrar AM, Jestaneiah S, Al-Serhani AM (2004). Regression of aneurysmal dilatations in a case of idiopathic retinal vasculitis, aneurysms and neuroretinitis (IRVAN) associated with allergic fungal sinusitis. Eye.

[56] Owens SL, Gregor ZJ (1992). Vanishing retinal arterial aneurysms: A case report. Br J Ophthalmol.

[57] Sashihara H, Hayashi H, Oshima K (1999). Regression of retinal arterial aneurysms in a case of idiopathic retinal vasculitis, aneurysms, and neuroretinitis (IRVAN). Retina.

[58] Tomita M, Matsubara T, Yamada H, Takahashi K, Nishimura T, Sho K (2004). Long term follow up in a case of successfully treated idiopathic retinal vasculitis, aneurysms, and neuroretinitis (IRVAN). Br J Ophthalmol.

[59] Rothova A, Lardenoye C (1998). Arterial macroaneurysms in peripheral multifocal chorioretinitis associated with sarcoidosis. Ophthalmology.

[60] Verougstraete C, Snyers B, Leys A, Caspers-Velu LE (2001). Multiple arterial ectasias in patients with sarcoidosis and uveitis. Am J Ophthalmol.

[61] Yamanaka E, Ohguro N, Kubota A, Yamamoto S, Nakagawa Y, Tano Y (2004). Features of retinal arterial macroaneurysms in patients with uveitis. Br J Ophthalmol.

[62] Ito Y, Nakano M, Kyu N, Takeuchi M (1976). Frosted branch angiitis in a child. Jpn J Clin Ophthalmol.

[63] Kleiner RC, Kaplan HJ, Shakin JL, Yannuzzi LA, Crosswell HH, McLean WC (1988). Acute frosted retinal periphlebitis. Am J Ophthalmol.

[64] Kleiner RC (1997). Frosted branch angiitis: Clinical syndrome or clinical sign?. Retina.

[65] Sykes SO, Horton JC (1997). Steroid-responsive retinal vasculitis with a frosted branch appearance in Crohn's disease. Retina.

[66] Markomichelakis NN, Barampouti F, Zafirakis P, Chalkiadakis I, Kouris T, Ekonomopoulos N (1999). Retinal vasculitis with a frosted branch angiitis-like response due to herpes simplex virus type 2. Retina.

[67] Farrardo J, Fonollosa A, Segura A, Garcia-Arumi J (2008). Frosted branch angiitis associated with Epstein-Barr virus sytemic infection. Ocul Immunol Inflamm.

[68] Sugin SL, Henderly DE, Friedman SM, Jampol LM, Doyle JW (1991). Unilateral frosted branch angiitis. Am J Ophthalmol.

[69] Borkowski LM, Jampol LM (1999). Frosted branch angiitis complicated by retinal neovascularization. Retina.

[70] Helm CJ, Holland GN (1993). Ocular tuberculosis. Surv Ophthalmol.

[71] Rosen PH, Spalton DJ, Graham EM (1990). Intraocular tuberculosis. Eye.

[72] Gupta A, Gupta V, Arora S, Dogra MR, Bambery P (2001). PCR-positive tubercular retinal vasculitis: Clinical characteristics and management. Retina.

[73] Biswas J, Sharma T, Gopal L, Madhavan HN, Sulochana KN, Ramakrishnan S (2002). Eales' disease: An update. Surv Ophthalmol.

[74] International Study Group for Behçet's Disease (1990). Criteria for diagnosis of Behçet's disease. Lancet.

[75] Verity DH, Wallace GR, Vaughan RW, Stanford MR (2003). Behçet's disease: From Hippocrates to the third millennium. Br J Ophthalmol.

[76] Arnold AC, Usaf M, Pepose JS, Foos RY (1984). Retinal periphlebitis and retinitis in multiple sclerosis: I: Pathologic characteristics. Ophthalmology.

[77] Lim JI, Tessler HH, Goodwin JA (1991). Anterior granulomatous uveitis in patients with multiple sclerosis. Ophthalmology.

[78] Graham EM, Francis DA, Sanders MD, Rudge P (1989). Ocular inflammatory changes in established multiple sclerosis. J Neurol Neurosurg Psychiatry.

[79] Engell T, Andersen PK (1982). The frequency of periphlebitis retinae in multiple sclerosis. Acta Neurol Scand.

[80] Birch MK, Barbosa S, Blumhardt LD, O'Brien C, Harding SP (1996). Retinal venous sheathing and the blood-retinal barrier in multiple sclerosis. Arch Ophthalmol.

[81] Rucker CW (1972). Sheathing of the retinal veins in multiple sclerosis: Review of pertinent literature. Mayo Clin Proc.

[82] Prineas JW, Wright RG (1978). Macrophages, lymphocytes, and plasma cells in the perivascular compartment in chronic multiple sclerosis. Lab Invest.

[83] Towler HM, Lightman S (2000). Symptomatic intraocular inflammation in multiple sclerosis. Clin Exp Ophthalmol.

[84] Rothova A (2000). Ocular involvement in sarcoidosis. Br J Ophthalmol.

[85] Nicholas J (2002). Sarcoidosis. Curr Opin Ophthalmol.

[86] DeRosa AJ, Margo CE, Orlick ME (1995). Hemorrhagic retinopathy as the presenting manifestation of sarcoidosis. Retina.

[87] Ohara K, Okubo A, Saski H (1995). Branch retinal vein occlusion in a child with ocular sarcoidosis. Am J Ophthalmol.

[88] Kimmel AS, McCarthy MJ, Blodi CF, Folk JC (1989). Branch retinal vein occlusion in sarcoidosis. Am J Ophthalmol.

[89] Duker JS, Brown GC, McNamara JA (1988). Proliferative sarcoid retinopathy. Ophthalmology.

[90] Siam AL, Meegan JM, Gharbawi KF (1980). Rift Valley fever ocular manifestations: Observations during the 1977 epidemic in Egypt. Br J Ophthalmol.

[91] Al-Hazmi A, Al-Rajhi AA, Abboud EB, Ayoola EA, Al-Hazmi M, Saadi R (2005). Ocular complications of Rift Valley fever outbreak in Saudi Arabia. Ophthalmology.

[92] Kaiser PK, Lee MS, Martin DA (2003). Occlusive vasculitis in a patient with concomitant West Nile virus infection. Am J Ophthalmol.

[93] Chan CK, Limstrom SA, Tarasewicz DG, Lin SG (2006). Ocular features of West Nile virus infection in North America: A study of 14 eyes. Ophthalmology.

[94] Teitelbaum BA, Newman TL, Tresley DJ (2007). Occlusive retinal vasculitis in a patient with West Nile virus. Clin Exp Optom.

[95] Herbort CP, Cimino L, Abu El-Asrar AM (2005). Ocular vasculitis: A multidisciplinary approach. Curr Opin Rheumatol.

